# Surgery for Acute Type A Aortic Dissection in A Pregnant Woman At 28 Weeks’ Gestation

**DOI:** 10.21470/1678-9741-2018-0225

**Published:** 2019

**Authors:** Yoshinori Kuroda, Tetsuro Uchida, Azumi Hamasaki, Atsushi Yamashita, Masahiro Mizumoto, Kentaro Akabane, Ai Ishizawa, Mitsuaki Sadahiro

**Affiliations:** 1 Division of Cardiovascular Surgery, Department of Surgery II, Yamagata University Faculty of Medicine, Yamagata-shi, Yamagata-ken, Japan.

**Keywords:** Aortic Valve, Cardiovascular Pregnancy Complications, Cardiovascular Surgical Procedures - Methods

## Abstract

A 27-year-old woman with sudden back pain was transported to our hospital. Abdominal ultrasonography revealed pregnancy of 28 weeks’ gestation. Computed tomography demonstrated a type A aortic dissection. Because of progressive fetal deterioration, an emergency cesarean section was forced to perform. The next day, simple hysterectomy followed by an aortic procedure was completed. Valve-sparing aortic replacement and total arch replacement were employed as central operations. The mother and baby are well 9 months postoperatively. Although the strategy for acute type A aortic dissection during pregnancy is controversial, collaborations among neonatologists, obstetricians, and cardiovascular surgeons can ensure mother and infant survival.

**Table:** 

Abbreviations, acronyms & symbols	
AAD	= Acute aortic dissection
CT	= Computed tomography
EF	= Ejection fraction

## Clinical Data

A 27-year-old woman with sudden back pain was transported to our hospital by ambulance. She had an abdominal protuberance and abdominal ultrasonography revealed that she was 28 weeks’ pregnant. Computed tomography (CT) was performed because she had uncontrollable severe back pain with hypertension; her blood pressure was 256/141 mmHg.

## Electrocardiography

Electrocardiography showed sinus rhythm with a heart rate of 83 beats/min, QRS axis of +57°, PR interval of 0.16 seconds, and overload in the left ventricle.

## Radiogram

Visceral *situs solitus* in levocardia was observed. There was also an increased cardiac area with a cardiothoracic index of 0.60 and no enlargement of the superior mediastinum.

## Echocardiography

Echocardiography showed normal ejection fraction (EF=63%) and wall thickening of the left ventricle. Aortic valve regurgitation, mitral valve regurgitation, and pericardial effusion were not observed.

## Diagnosis

**Fig. 1:**
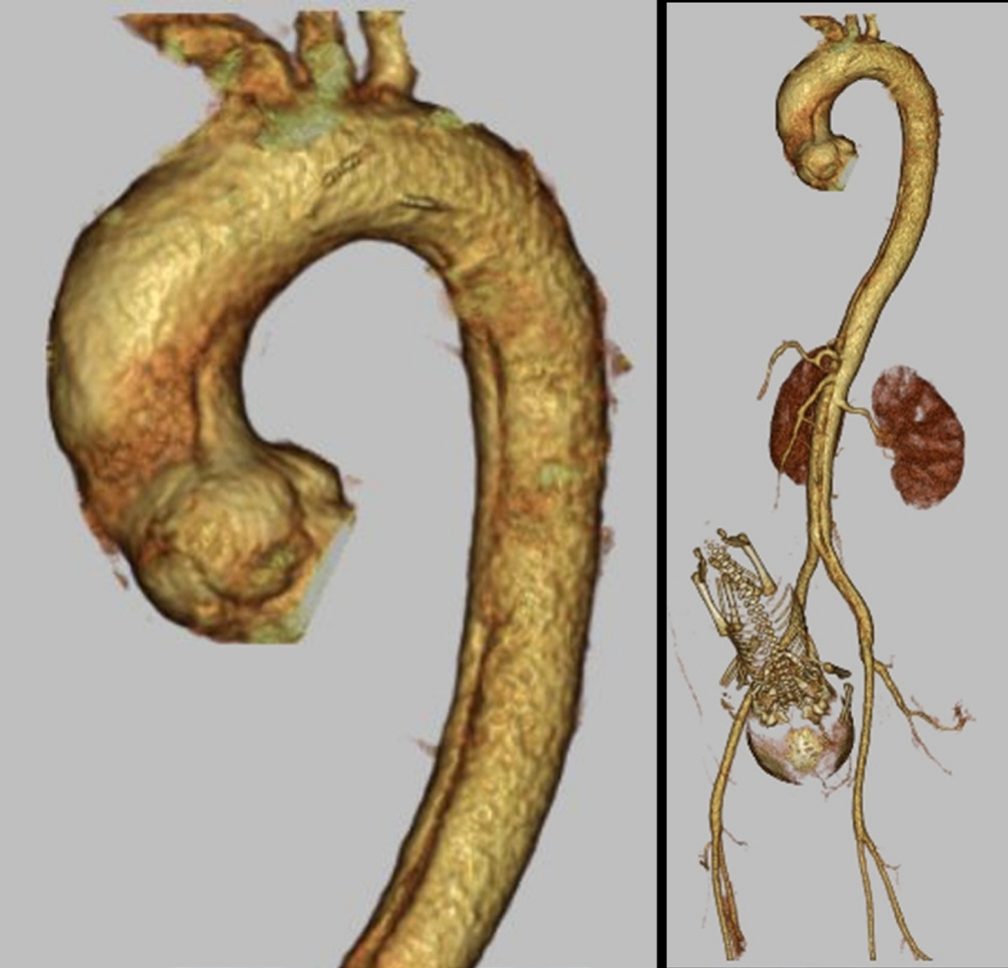
Preoperative computed tomography image Acute type A aortic dissection reaches the abdominal aorta, and pregnancy is confirmed.

CT revealed an acute aortic dissection (AAD), and the pelvic view showed a fetus (Figure 1). The size in diameter of ascending, arch, and descending aorta were 33mm, 29mm, and 23mm respectively. Antihypertensive therapy was immediately started after acute AAD was diagnosed. Delivery was considered possible according to the stage of fetal development. Therefore, we decided to perform an emergency cesarean section followed by surgery for acute AAD to rescue the mother and fetus.

## Operation

The emergency cesarean section was performed by obstetricians, with the patient under general anesthesia. The baby, weighing 756 g, was intubated immediately after delivery and transferred to the neonatal intensive care unit. On the next day of the cesarean section, advanced hysterectomy was conducted because there was a risk of major atonic bleeding uterus during aortic surgery. Median sternotomy was performed, and cardiopulmonary bypass was established through the right axillary artery, right femoral artery, and superior and inferior vena cava. After cardiac arrest, the ascending aorta was cut open. An intimal tear developed from just above the non-right commissure to beside the right coronary artery ostium. As the dissection extended to the aortic root, valve sparing aortic root replacement (the reimplantation procedure) and total arch replacement with frozen elephant trunk were performed.

**Fig. 2:**
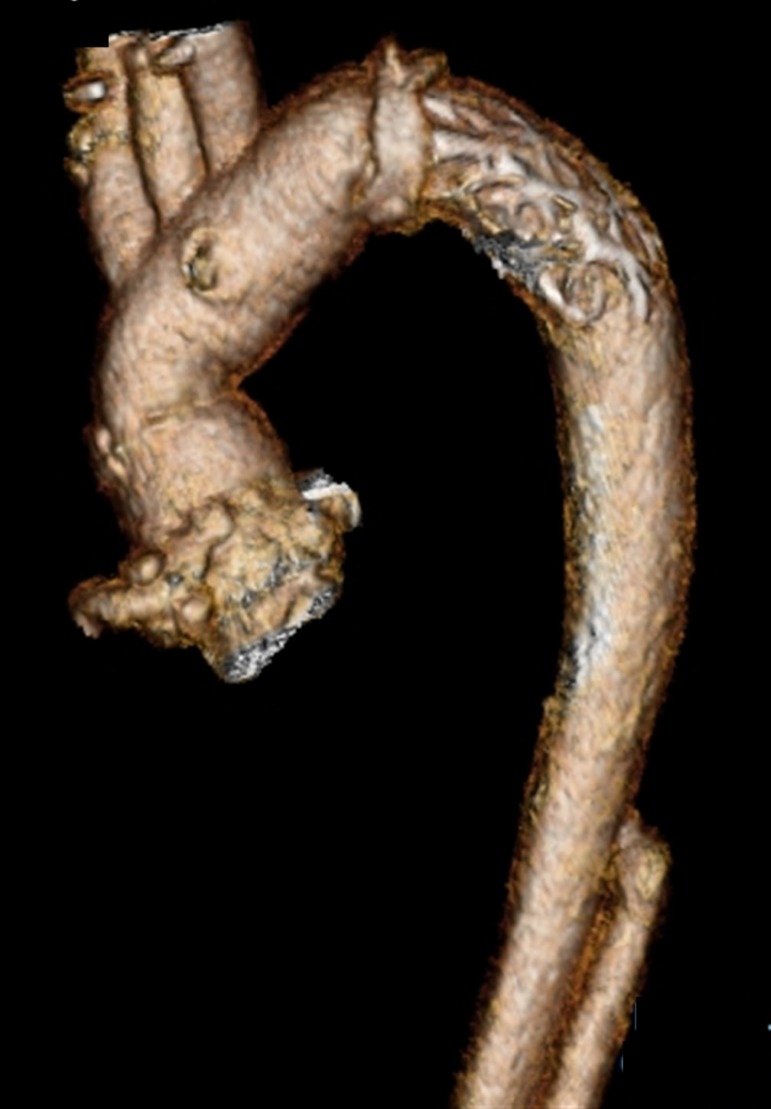
Postoperative computed tomography image Repair of the dissected aorta is shown with an artificial vascular graft.

Histopathological analysis showed no evidence of connective tissue disease. Postoperative CT demonstrated resolved AAD (Figure 2). The mother and baby were doing well 9 months postoperatively.

Acute AAD during pregnancy has been reported^[1,2]^. Acute AAD during pregnancy endangers the lives of both the mother and fetus. The treatment strategies for acute AAD during pregnancy are controversial. Treatment strategies are considered to include the following: aortic surgery after cesarean section, delivery after aortic surgery, and simultaneous cesarean section and aortic surgery. In situations wherein the fetus can endure delivery and the mother’s acute AAD is stable, a staged aortic surgery after cesarean section is possible. However, when acute AAD is unstable, there is no choice but to perform cesarean section and aortic surgery simultaneously. If fetal development is inadequate, aortic surgery must be performed before delivery.

It has been reported that the natural mortality of infants with extremely low birth weight during their neonatal intensive care unit stay is 8.3% at more than 28 weeks’ gestation. The mortality according to birth weight was less than 12% in infants weighing over 700 g^[3]^. In our case, the possibility of delivery was increased when the fetus was more than 28 weeks of gestation and weighed over 700 g.

Although the strategy for acute AAD during pregnancy is still controversial, our strategy for the present case saved the mother and baby.

**Table:** 

Authors' roles & responsibilities	
YK	Substantial contributions to the conception or design of the work; or the acquisition, analysis, or interpretation of data for the work; drafting the work or revising it critically for important intellectual content; final approval of the version to be published
TU	Substantial contributions to the conception or design of the work; or the acquisition, analysis, or interpretation of data for the work; drafting the work or revising it critically for important intellectual content; final approval of the version to be published
AH	Substantial contributions to the conception or design of the work; or the acquisition, analysis, or interpretation of data for the work; final approval of the version to be published
AY	Substantial contributions to the conception or design of the work; or the acquisition, analysis, or interpretation of data for the work; final approval of the version to be published
MM	Substantial contributions to the conception or design of the work; or the acquisition, analysis, or interpretation of data for the work; final approval of the version to be published
KA	Substantial contributions to the conception or design of the work; or the acquisition, analysis, or interpretation of data for the work; final approval of the version to be published
AL	Substantial contributions to the conception or design of the work; or the acquisition, analysis, or interpretation of data for the work; final approval of the version to be published
MS	Substantial contributions to the conception or design of the work; or the acquisition, analysis, or interpretation of data for the work; final approval of the version to be published
